# Case Report: Successful fetoscopic release of pseudoamniotic bands in twin-to-twin transfusion syndrome and twin reversed arterial perfusion sequence: report of two rare cases and review of the literature

**DOI:** 10.3389/fmed.2026.1829018

**Published:** 2026-07-17

**Authors:** Luyao Li, Aiqing Zhang, Xueju Wang, Pengbo Yuan, Chengqing Hu, Yangyu Zhao, Yuan Wei

**Affiliations:** 1 Department of Obstetrics and Gynecology, Peking University Third Hospital, Beijing, China; 2 National Clinical Research Centre for Obstetrics and Gynaecology, Beijing, China

**Keywords:** fetoscopic release, monochorionic pregnancy, pseudoamniotic band syndrome, twin reversed arterial perfusion sequence, twin-to-twin transfusion syndrome

## Abstract

**Background:**

Pseudoamniotic band syndrome (PABS) is an iatrogenic amniotic disruption sequence that may occur after invasive fetal procedures, including fetoscopic laser therapy and amniocentesis. Fetoscopic release of the bands has been reported in singleton pregnancies, but it remains rare in twin pregnancies after intrauterine fetal surgery. This study aimed to report two rare cases of PABS occurring after invasive fetal interventions-one for twin-to-twin transfusion syndrome (TTTS) and one for twin reversed arterial perfusion (TRAP) sequence-both of which were successfully managed by fetoscopic band release, and to review the clinical characteristics of PABS in TTTS.

**Methods:**

We describe one rare case off fetal abdominal constriction caused by amniotic bands following TTTS fetoscopic surgery, and one case of PABS in TRAP sequence after microwave ablation and amniocentesis. Both cases were successfully treated *in utero* via subsequent fetoscopic band release later. A literature review was conducted on PABS cases after TTTS intervention, comparing cases managed expectantly versus those treated with fetoscopic release.

**Results:**

We reported two cases of PABS with constriction of the fetal abdomen and ankle, respectively, treated by fetoscopic band release, and both cases achieved successful outcomes after fetoscopic band release. A literature review identified 50 reported PABS cases after TTTS treatment. PABS occurred predominantly in recipients (78.0%, 39/50 cases), primarily affecting fetal limbs (88.2%, 45/51 fetuses). Antenatal detection was low (20.0%, 10/50 cases), and without intervention, 9.5% (4/42 fetuses) developed fetal limb amputation. Including our case, only 8 PABS cases in TTTS have undergone fetoscopic release, with a median interval of 4.3 weeks post-TTTS fetoscopic laser. The median GA at fetoscopic release surgery was 23.5 (range, 21–27.4) weeks, with a median interval of 5.6 (range, 0.8–11.9) weeks between fetoscopic release and delivery (at 30.7 weeks; range, 24.7–34.9). Of these, 37.5% (3/8) of the newborns required further plastic surgery after birth, but all fully recovered functionally without amputation.

**Conclusion:**

Serial ultrasound surveillance after fetal interventions should include PABS assessment, particularly 4 weeks post-procedure. Although antenatal diagnosis remains challenging, fetoscopic band release appears technically feasible in twin pregnancies and potentially beneficial in carefully selected, antenatally diagnosed cases, but the evidence remains limited and vulnerable to publication bias.

## Introduction

Twin-to-twin transfusion syndrome (TTTS) and twin reversed arterial perfusion (TRAP) sequence are two specific complications of monochorionic twin pregnancies accounting for 10–15% and 1% of monochorionic (MC) twin pregnancies, respectively (1). Owing to the superficial vascular anastomoses of the monochorionic placenta serving as the anatomical basis of TTTS, fetoscopic laser coagulation is the first-line treatment. TRAP sequence is characterized by the presence of an acardiac mass perfused by the normal pump twin, so the treatments aim at occluding blood flow to the acardiac twin, including microwave ablation, fetoscopic laser coagulation, and so on (2, 3).

Amniotic band syndrome (ABS) is a spectrum of congenital anomalies that have the presence of amniotic bands that become entangled around the fetus or umbilical cord. Spontaneous ABS is generally considered a congenital amniotic disruption sequence and may arise without a preceding invasive procedure. Pseudoamniotic band syndrome (PABS), in contrast, is an acquired or iatrogenic form that develops after fetal membrane disruption related to invasive intrauterine procedures such as fetoscopy, septostomy, cord occlusion, or amniocentesis (4).

After FLP for TTTS, the reported prevalence of PABS is approximately 1.8–3.3% (4–7). Amniotic bands can compress different fetal body parts, potentially leading to fetal ischemia, edema, limb amputation, structural defects, or even fetal demise (4). Fetoscopic release of the bands has been reported in some amniotic band syndrome cases (ABS) occurring spontaneously in singleton pregnancy and in a small number of post-intervention PABS cases, using various lysis techniques, including scissors dissection or laser coagulation (5, 8–12).

Here, we present one rare case of abdominal constrictions by amniotic bands following TTTS fetoscopic surgery, and one case of PABS in TRAP sequence after microwave ablation and amniocentesis. Both cases were successfully treated *in utero* by fetoscopy later. Since only a few cases occurred in TRAP sequence, we only summarized all published TTTS-associated PABS cases, to investigate the clinical features, natural history, risk factors and outcomes.

## Methods

### Case report

TTTS was diagnosed in MC pregnancies using standard amniotic-fluid criteria: before 20 weeks, the maximum vertical pocket (MVP) of the recipient fetus was ≥ 8 cm, while the MVP of the donor fetus was ≤ 2 cm; after 20 weeks, the MVP of the recipient fetus was ≥ 10 cm, while the MVP of the donor fetus was ≤ 2 cm. The stages of TTTS were determined according to Quintero criteria, as follows: stage I: the bladder of the donor fetus is visible; stage II: the bladder of the donor fetus is no longer visible; stage III: the presence of any fetus showing abnormal blood flow; stage IV: recipient fetal edema; and stage V: any or all fetuses are dead (13).

Surgical procedures were performed after multidisciplinary discussion including fetal surgeons, a pediatrician and a maternal-fetal medicine specialist, counseling with the parents, and after obtaining the patient’s informed consent. Diluted lidocaine was administered for local anesthesia of the skin and subcutaneous tissue. Intraoperative antibiotic prophylaxis with 1 g cefazolin was administered intravenously. All fetoscopic procedures were performed by the same experienced physician in fetal medicine. Fetoscopic laser surgery was performed using a 1.3-mm fetoscope (Storz, Germany) and a 400-μm Nd-YAG laser fiber (Dornier MedTech, Germany) via an 8-Fr cannula. The fetoscopic amniotic band release procedure was performed through a single uterine entry using a 1.3-mm fetoscope with scissors via an 8-Fr catheter. An amnioinfusion of saline solution was performed to improve visualization during the procedure.

### Literature review

We searched PubMed using the following Medical Subject Headings (MeSH) and keywords: amniotic band syndrome, fetoscopy, fetal surgery, pseudoamniotic band syndrome, and fetofetal transfusion. Reports were included if they were published in English, described PABS after TTTS-related fetal intervention, and provided sufficient clinical details. Data on the stage of TTTS, the gestational age at diagnosis and surgery of TTTS, the gestational age at diagnosis and surgery of PABS, the affected fetal limbs, umbilical cord involvement, perioperative complications, time of birth, and outcomes were collected.

## Results

### Case 1: PABS with abdominal constriction after FLP for TTTS

A 35-year-old woman, gravida 2, para 1, with monochorionic diamniotic twin pregnancy, was referred to our fetal medicine center at 18 weeks of gestation for stage II TTTS. Ultrasound showed polyhydramnios in the recipient sac (MVP = 12.3 cm) and anhydramnios in the donor sac (MVP = 0 cm) with an absent donor bladder. After counseling, the parents accepted FLP of placental vascular anastomoses at 18 + 3 weeks without immediate maternal or fetal complications.

**FIGURE 1:**
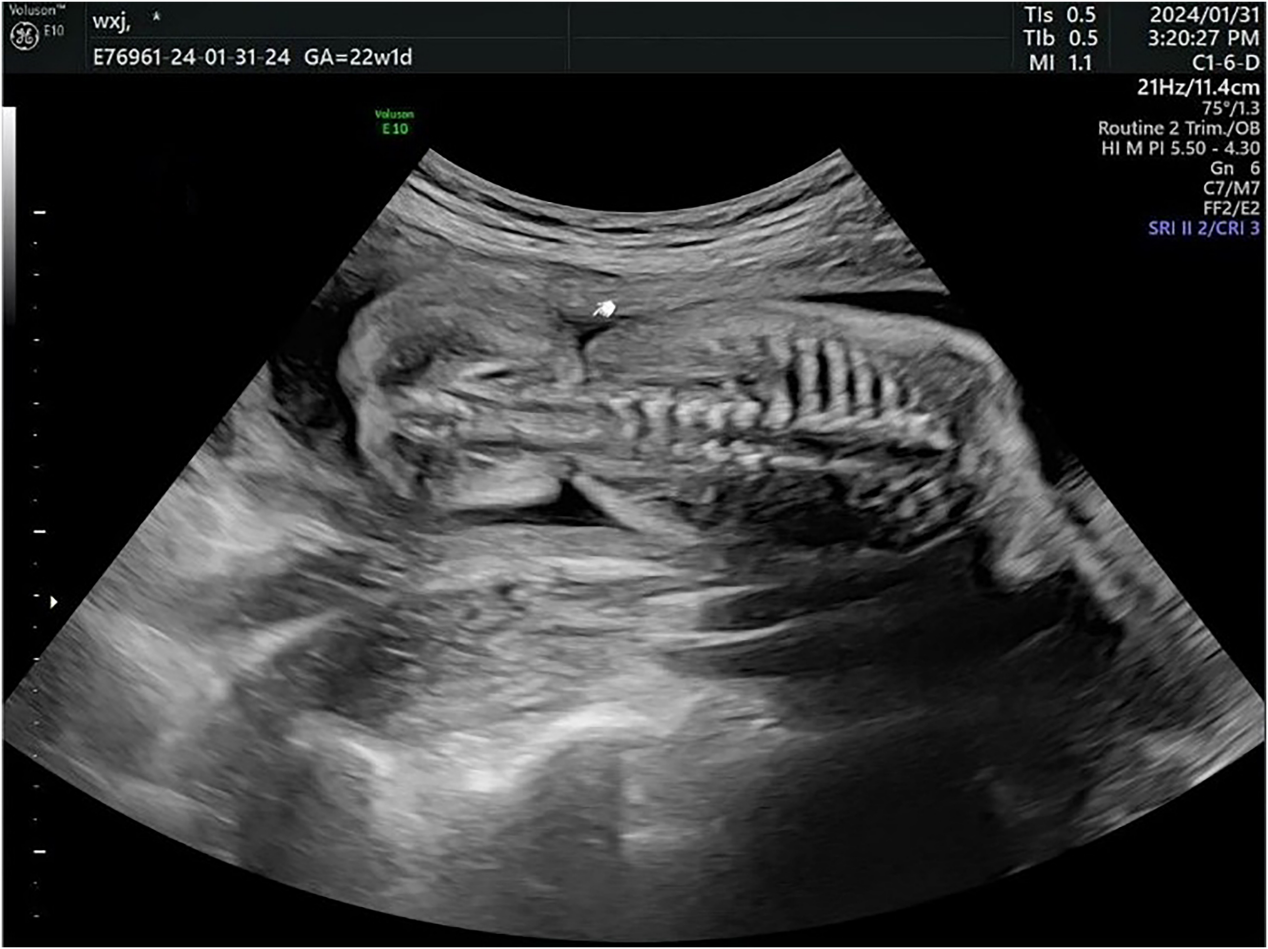
Ultrasonographic image of the fetal abdomen. The patient was a 35-year-old woman with twin-to-twin transfusion syndrome (TTTS) following fetoscopic laser photocoagulation (FLP). At 22 + 5 weeks of gestation, ultrasonography revealed amniotic bands wrapped around the recipient twin, with severe constriction of the abdomen.

The patient had regular ultrasound examination postoperatively and both twins were alive with concordant MVP, visible bladders and normal umbilical artery blood flow. However, 4 weeks postoperatively (at 22 + 5 weeks of gestation), ultrasound revealed amniotic bands encircling the recipient twin, with marked constriction at the abdominal level (Figure 1).

**FIGURE 2:**
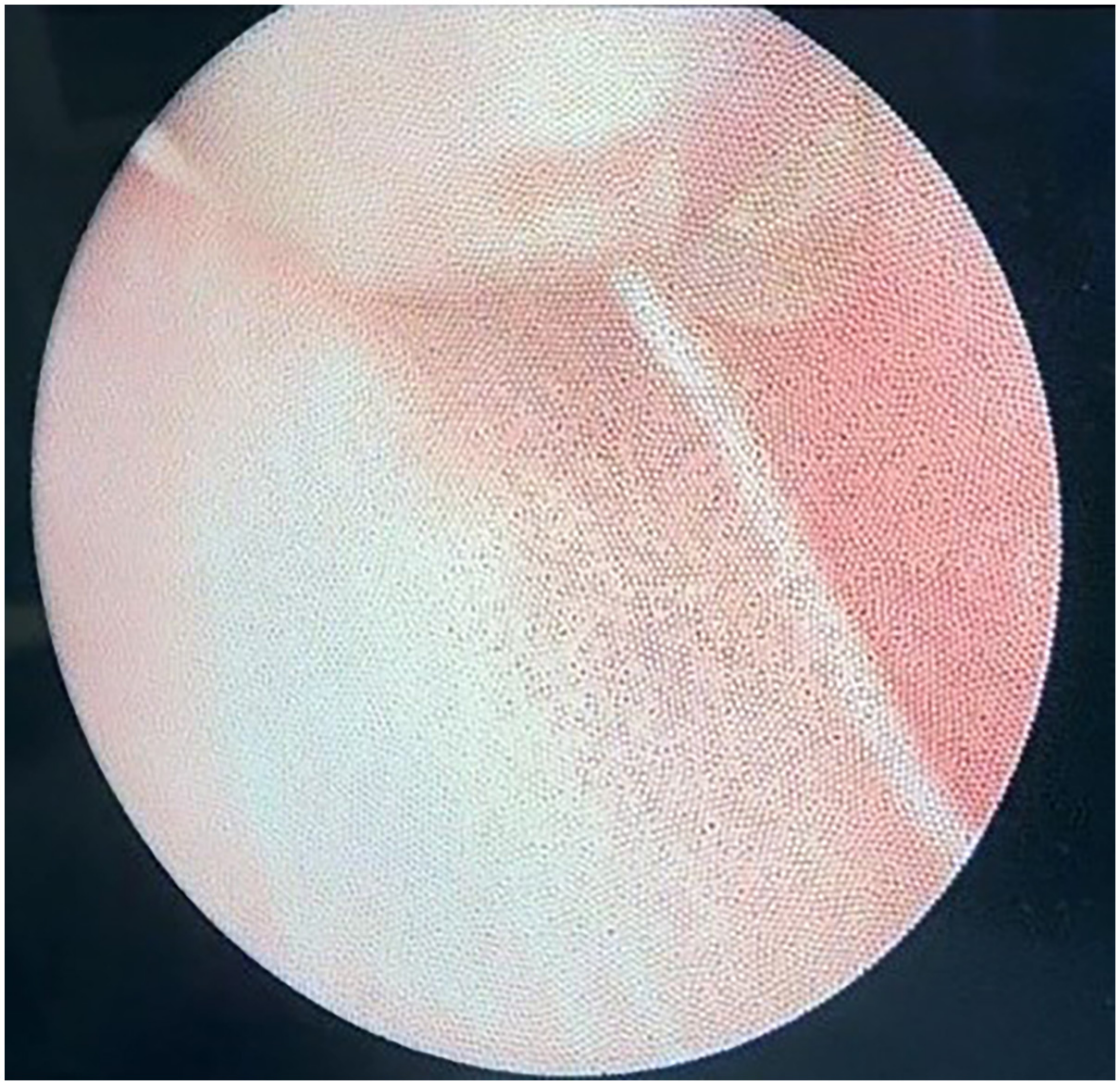
Intraoperative fetoscopic image of an amniotic band wrapped around the fetal abdomen. Fetoscopic release of the amniotic band was performed at 23 weeks’ gestation via a single-trocar approach. Fetoscopy demonstrated a tight constricting band encircling the fetal abdomen.

Because of concern for possible fetal compromise, fetoscopic release of amniotic bands was performed at 23 weeks by one-trocar approach. Fetoscopy revealed floating amniotic membranes around the recipient fetus and a tight band encircling the abdomen (Figure 2). The amniotic bands around the fetal abdomen were divided by scissors successfully and immediate loosening of the constriction was observed after band division. Most remaining floating amniotic bands were released indirectly by dissecting them away from the umbilical cord using scissors. No intraoperative maternal or fetal complications occurred. Follow-up ultrasound showed persistent abdominal indentation but no progression of constriction, and fetal growth remained appropriate.

**FIGURE 3:**
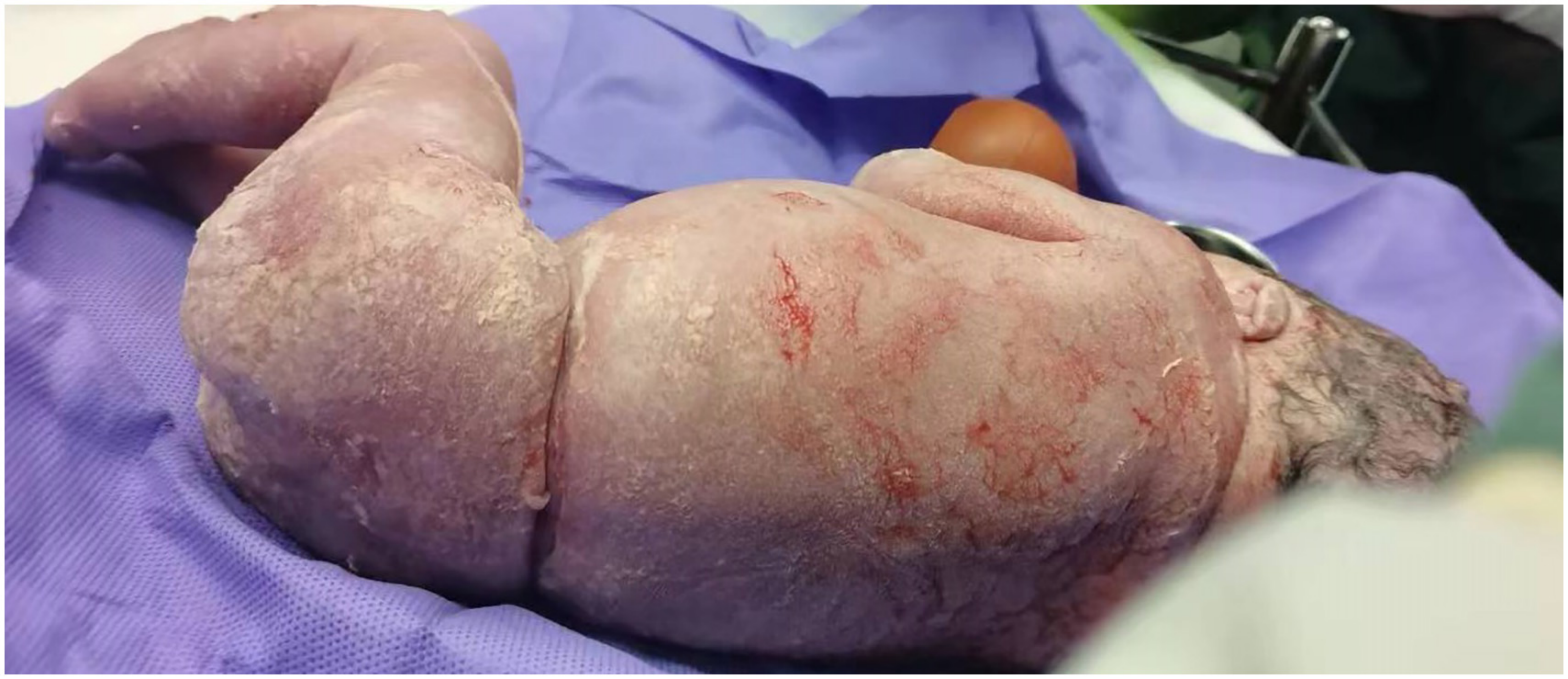
Postnatal photograph of the recipient twin. The twins were delivered at 34 + 6 weeks of gestation. The birth weight of the recipient was 2,260 g, and that of the donor was 2,090 g. A residual abdominal constriction ring with skin laceration below the umbilicus was observed in the recipient.

**FIGURE 4:**
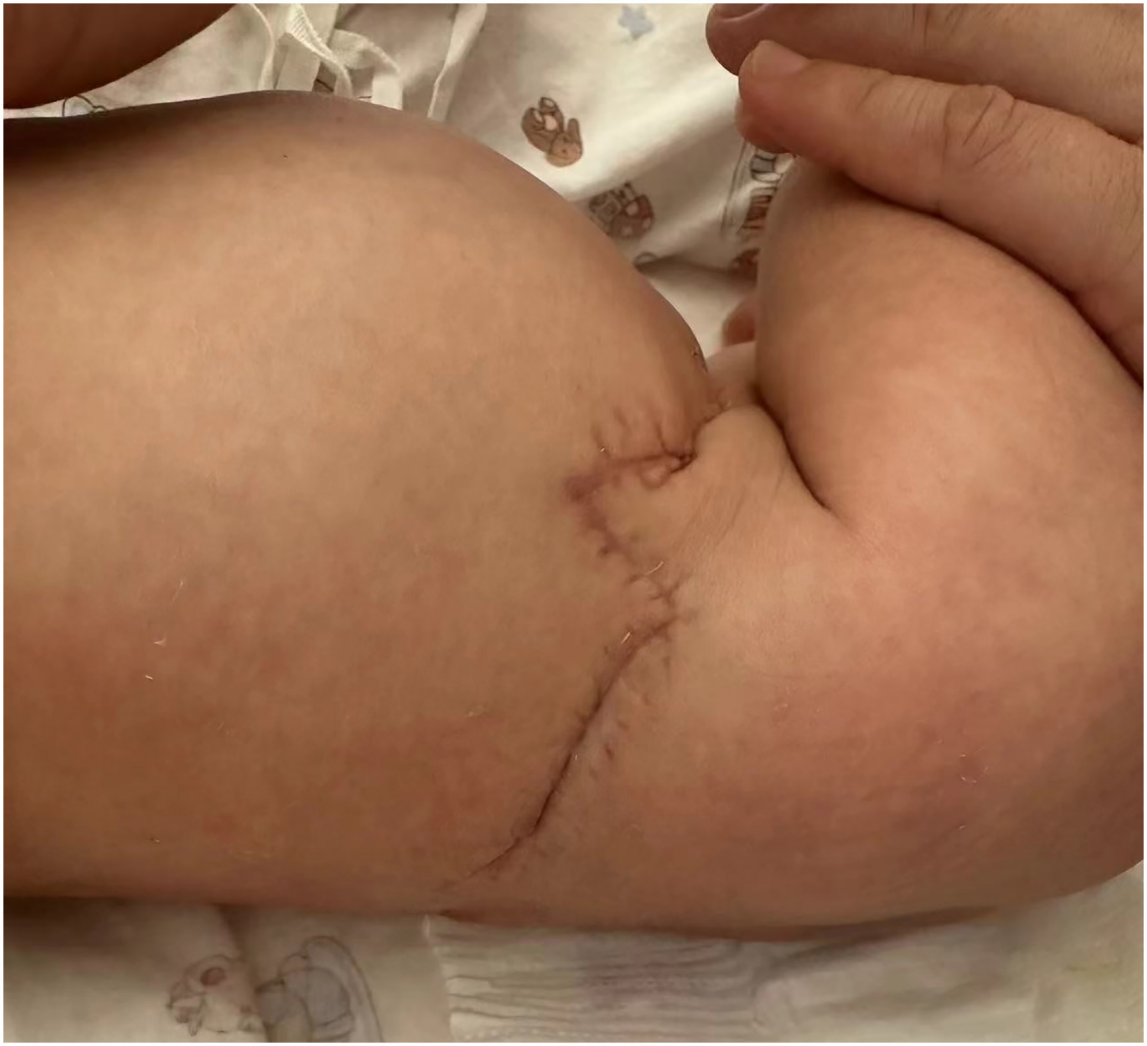
Postnatal photograph of the affected infant. Abdominal Z-plasty was performed, with satisfactory functional and cosmetic outcomes at 1 month postoperatively.

At 34 + 6 weeks’ gestation, preterm premature rupture of membranes (PPROM) occurred, and an emergency cesarean delivery was performed.. Two male neonates were delivered with 1- and 5-min Apgar scores of 8 and 10, respectively. Birth weights were 2,260 g for the recipient twin and 2,090 g for the donor twin.. The recipient presented with a residual abdominal constriction ring with skin laceration below the umbilicus (Figure 3). Pathological examination of the placenta demonstrated a laser coagulation line and no residual anastomosis. The affected infant underwent surgical removal of the residual band ring and accepted a surgical repair of the abdomen by Z-plasty. Functional and morphological recovery of the abdomen was confirmed at 1 month (Figure 4).

### Case 2: PABS with right ankle constriction after treatment of TRAP sequence

A 32-year-old woman had a monochorionic triamniotic triplet pregnancy complicated by TRAP sequence with an acardiac fetus (Fetus C), diagnosed at 17 weeks. Microwave ablation of the acardiac fetus was performed at 19 + 1 weeks, and amniocentesis was conducted d for the structurally normal Fetus A during the same procedure.

**FIGURE 5:**
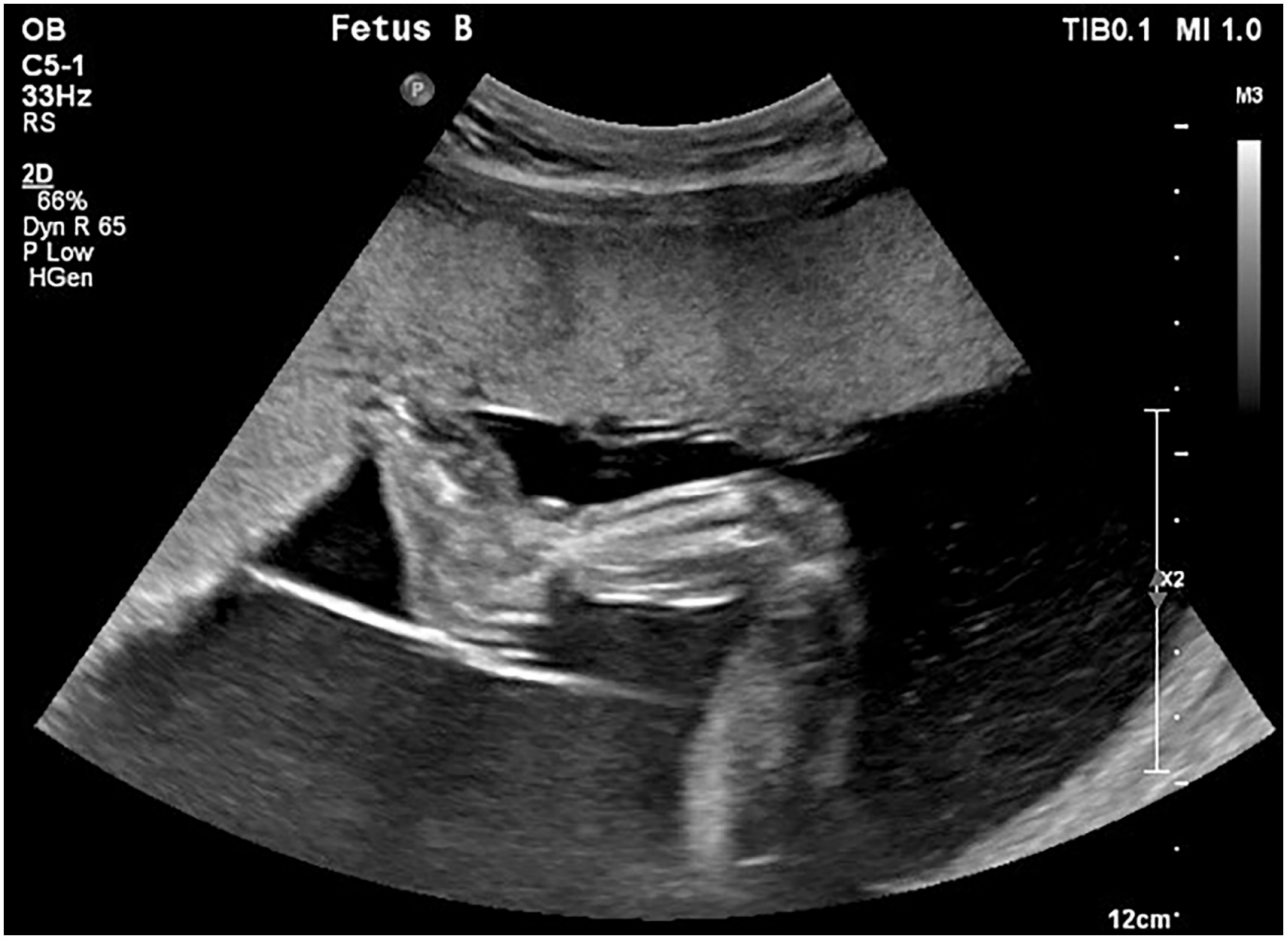
Ultrasonographic image of the fetal right foot. The 32-year-old mother had twin reversed arterial perfusion (TRAP) sequence and received microwave ablation for the acardiac fetus. Ultrasonography demonstrated an amniotic band encircling the fetal ankle, associated with subcutaneous tissue edema thickened to 0.6 cm.

**FIGURE 6:**
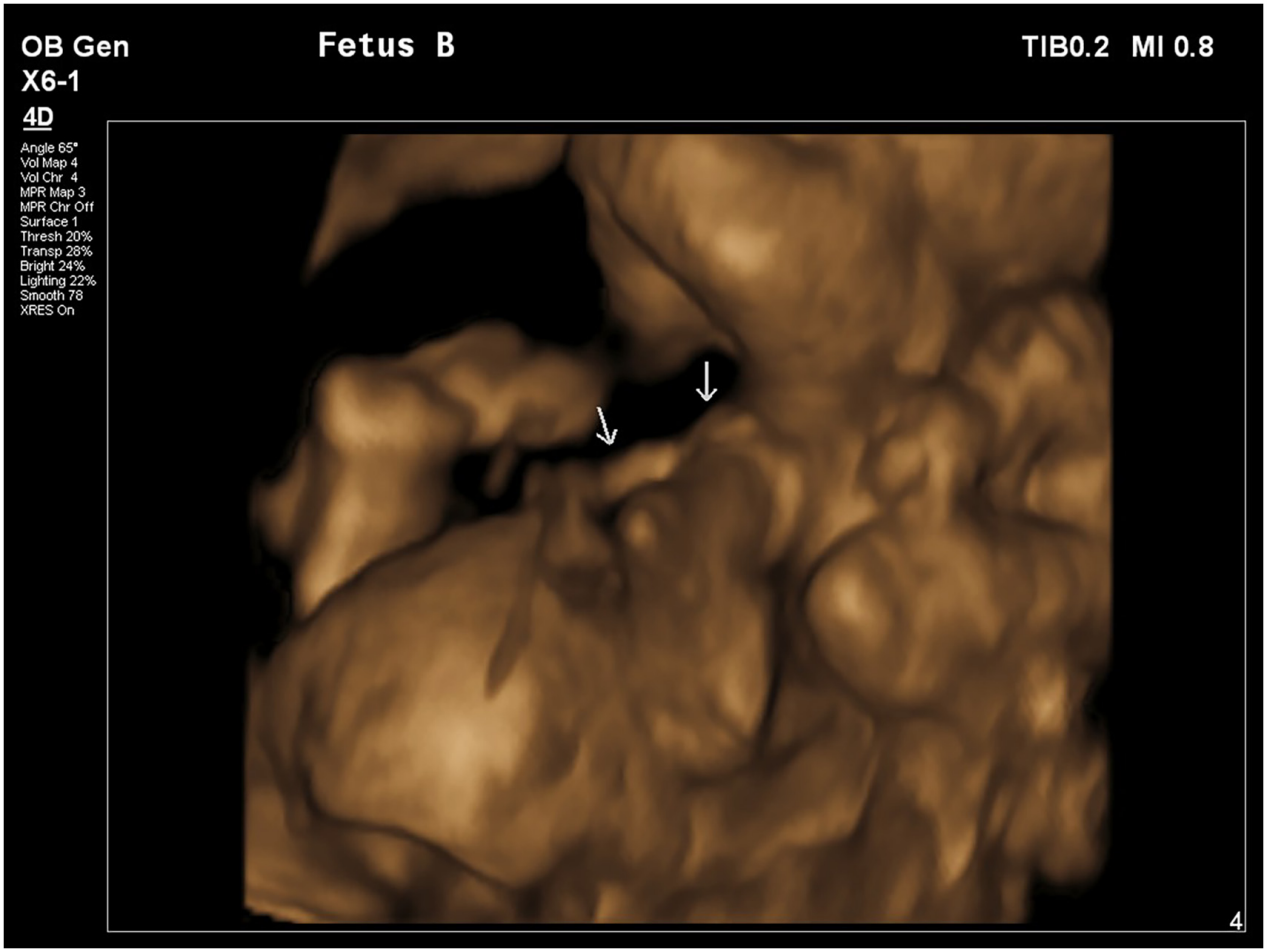
Three-dimensional ultrasound image of the fetus. The white arrow indicates the fetal right ankle, with faint umbilical cord signs visible around it.

**FIGURE 7:**
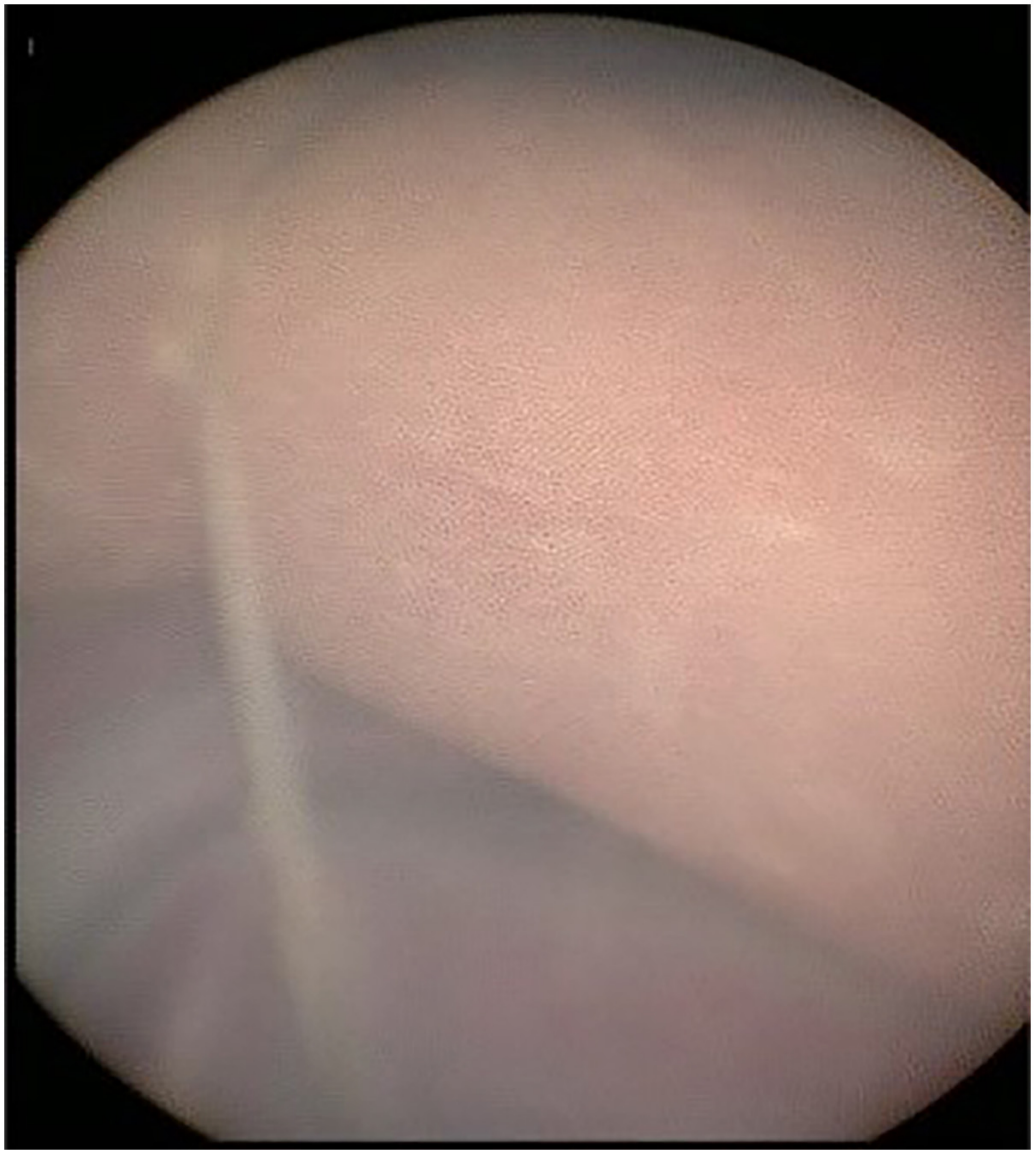
Intraoperative fetoscopic image of an amniotic band wrapped around the fetal right ankle. Fetoscopic release of pseudoamniotic bands was performed at 25 weeks of gestation, and amniotic bands wrapped around the fetal right ankle were visualized intraoperatively.

The patient was referred to our center after ultrasound showed an amniotic band constricting the right ankle of Fetus A, accompanied by marked distal edema (Figure 5). The subcutaneous soft tissue thickness of the fetal right foot was 0.6 cm, and faint umbilical cord echoes were detected around the ankle (Figure 6). After extensive counseling, the patient underwent fetoscopic pseudoamniotic band release at 25 weeks (Figure 7). Amniotic bands were divided with scissors successfully and removed from the uterine cavity with forceps. Postoperative ultrasound monitoring revealed alleviated fetal pedal edema, with the subcutaneous soft tissue thickness reduced to 0.4 cm. The pregnancy continued until 34 weeks. Neonatal examination showed no evident constriction or functional impairment of the right foot.

### Review results

From November 2000 to January 2026, the review included 50 TTTS- associated PABS cases from 15 articles: 42 cases managed without intrauterine release and 8 cases treated with fetoscopic release, including Case 1. Detailed case-level data are presented in Supplementary Tables 1, 2, and a summary is provided in Table 1.

**TABLE 1:** Summary of TTTS-associated PABS cases identified in the focused review.

Variable	Without intrauterine release	With fetoscopic release
Cases	42	8
Affected fetuses	43	9
TTTS stage	Available 23/42 cases; Stage I: 17.4% (4/23 cases) Stage II: 30.4% (7/23 cases) Stage III: 43.5% (10/23 cases) Stage IV: 8.7% (2/23 cases)	Available 7/8 cases; Stage II: 28.6% (2/7 cases) Stage III: 71.4% (5/7 cases)
Placental location	Available 11/42 cases; Posterior: 54.5% (6/11 cases) Anterior: 45.5% (5/11 cases)	Available 2/8 cases; Posterior: 100% (2/2 cases)
GA at TTTS intervention	Available 40/42 cases; Median 17.7 weeks (IQR, 16.6–19.2)	Available 7/8 cases; Median 18.0 weeks (range, 16.6–18.9)
Timing of PABS diagnosis	Available 42/42 cases; Postnatal: 95.2% (40/42 cases) Antenatal: 4.8% (2/42 cases)	Available 5/8 cases; Median 22.0 weeks (range, 21.0–25.1)
Affected fetus	Available 42/42 cases; Recipient: 71.4% (30/42 cases) Donor:26.2% (11/42 cases) Both twins: 2.4% (1/42 cases)	Available 8/8 cases; Recipient: 87.5% (7/8 cases) Both twins: 12.5% (1/8 cases)
Affected body part	Available 42/43 fetuses; Upper limbs: 26.2% (11/42 fetuses) Lower limbs: 54.8% (23/42 fetuses) Both limbs: 9.5% (4/42 fetuses) Umbilical cord only: 4.8% (2/42 fetuses) Face: 2.4% (1/42 fetuses) Trunk: 2.4% (1/42 fetuses)	Available 9/9 fetuses Fetal limbs: 77.8% (7/9 fetuses) Umbilical cord only: 11.1% (1/9 fetuses) Abdomen: 11.1% (1/9 fetuses)
Amputation	Available 42/43 fetuses; 9.5% (4/42 fetuses)	Available 9/9 fetuses 0
Cord involvement	Available 33/42 fetuses; 15.2% (5/33 fetuses)	Available 9/9 fetuses 66.7% (4/6 fetuses)
PPROM	Available 37/42 cases; 32.4% (12/37 cases)	Available 6/8 cases; 66.7% (4/6 cases)
IUFD	Available 42/42 cases; Total IUFD: 42.9% (18/42 cases) Recipient IUFD: 9.5% (4/42 cases) Donor IUFD: 28.6% (12/42 cases) Both IUFD: 4.8% (2/42 cases)	Available 8/8 cases; Donor IUFD: 37.5% (3/8 cases)
GA at delivery	Available 40/42 cases; Median 31.0 weeks (IQR, 29.3–33.5)	Available 8/8 cases; Median 30.7 weeks (range, 24.7–34.9)
Postnatal interventions	Available 11/42 cases; Several cases required reconstructive care or had digital amputation/necrosis	Available 8/8 cases; 3/8 newborns required plastic/reconstructive procedures; one reported reduced gripping function

GA, gestational age; IUFD, intrauterine fetal demise; PABS, pseudoamniotic band syndrome; PPROM, preterm premature rupture of membranes; TTTS, twin-to-twin transfusion syndrome.

### PABS in TTTS without intrauterine release

Among 42 TTTS-associated PABS cases without intrauterine release (Supplementary Table 1), 41/42 (97.6%) occurred after FLP and 1/42 (2.4%) developed after selective fetal reduction via bipolar cord coagulation. TTTS stage was reported in 23/42 cases (54.8%); stage III was most common (10/23, 43.5%), followed by stage II (7/23, 30.4%) and stage I (4/23, 17.4%). Placental location was reported in only 11/42 cases (26.2%); 6 cases were posterior and 5 were anterior. GA at TTTS intervention was available in 40/42 cases and the median was 17.7 weeks (IQR, 16.6–19.2). Timing of PABS detection was reported for all 42 cases, but almost all diagnoses were made after birth (40/42, 95.2%); only 2/42 cases (4.8%) were antenatally recognized. The affected fetus was the recipient in 30/42 cases (71.4%), the donor in 11/42 (26.2%), and both twins in 1/42 (2.4%).

The affected body part was reported in 42/43 fetuses. Almost all affected fetuses (38/42, 90.5%) had extremity involvement. Lower limbs were most frequently affected, accounting for 54.8% (23/42 fetuses), followed by upper limbs in 26.2% (11/42 fetuses), and both upper and lower limbs in 9.5% (4/42 fetuses). The following fetal extremities were most affected: foot (19/42 fetuses, 45.2%), arm (10/42 fetuses, 23.8%), leg (7/42 fetuses, 16.7%), hand (6/42 fetuses, 14.3%), and ankle (3/42 fetuses, 7.1%). In 4 of the 42 fetuses (9.5%), amputation of limbs or toes occurred.

The survival rate of at least one fetus is 95.2% (40/42 cases). The dual twin survival rate was 57.1% (24/42 cases), and the single fetal survival rate (i.e., only one fetus survived) was 38.1% (16/42 cases). Most single intrauterine deaths occurred in donor fetus (75.0%, 12/16 cases). PPROM occurred in 32.4% (12/37 cases) of the cases. 40 cases reported gestational weeks (GA) at delivery, and median GA was 31.0 weeks (IQR, 29.3–33.5). Among cases with fetal survival (available in 38/40 cases), preterm birth (PTB) occurred in 94.7% (36/38 cases), remaining the major problem in TTTS cases. The incidence rates of PTB < 34 weeks, < 32 weeks, < 28 weeks were 76.3% (29/38 cases), 55.3% (21/38 cases), and 13.2% (5/38 cases), respectively.

### PABS in TTTS with fetoscopic release

There were only five previous published articles and 8 reported cases including Case 1 of PABS in TTTS after FLP following fetoscopic release of amniotic bands (Supplementary Table 2). GA at FLP was documented in 7/8 cases, with a median of 18.0 weeks (range, 16.6–18.9). Timing of antenatal PABS diagnosis was available in 5/8 cases; the median was 22.0 weeks (range, 21.0–25.1), corresponding to a median interval of 4.3 weeks after FLP. Fetoscopic release was performed at a median GA of 23.5 weeks (range, 21.0–27.4), with a median interval of 5.6 (range, 0.8–11.9) weeks between fetoscopic release and delivery (median 30.7 weeks, range from 24.7 to 34.9). The affected fetus was the recipient in 7/8 cases and both twins in 1/8. Among 9 affected fetuses, 7 (77.8%) had limb involvement, 1 had abdominal involvement (Case 1), and 1 had isolated umbilical cord involvement. The technique of fetoscopic release of amniotic bands described in the literature included one using YAG laser fiber only, one using YAG laser fiber with scissors, and six using scissors or forceps. The dual survival rate was 62.5% (5/8 cases). 37.5% (3/8 cases) of the newborns needed further plastic surgery after birth but all of them fully recovered functionally without amputation.

## Discussion

To date, 50 reported cases of PABS after intrauterine intervention for TTTS have been reported in the literature. The presumed mechanism of PABS differs from spontaneous ABS (14, 15). In TTTS with PABS cases, most affected twins were recipient (78.0%, 39/50), which was consistent with many research (6, 16). In TTTS, the recipient sac is the operative space during FLP, and recipient polyhydramnios may allow greater movement of disrupted membrane strands, which may explain the predominance of recipient involvement. Iatrogenic uterine access may lead to traumatic fetal membrane disruption, chorioamniotic membrane separation (CMS), or septostomy. This ruptured, floating amniotic membrane may entrap the fetal extremities and umbilical cord in both twins, resulting in PABS.

In the cases without treatment of TTTS-associated PABS, only 4.8% cases were antenatally recognized. Increased awareness for antenatal ultrasound detection of PABS is warranted. Ultrasound surveillance after fetal intervention should therefore include active assessment for floating membranes, constriction rings, distal limb edema, abnormal distal perfusion, restricted fetal movement, and cord entanglement.

CMS was observed in nearly 40% of patients with ABS (17) and in approximately 20% of TTTS cases after FLP (18–20). Knijnenburg et al. (4) reported CMS in 93% of pregnancies complicated by PABS, compared to 25% in those without PABS. A lower gestational age at laser surgery was identified as a predictor for CMS (4, 18) and for TTTS after FLP with PABS (4). The median gestational age at FLP was 20.0 (IQR, 17.7–22.0) weeks in TTTS without PABS (4), while we found that of these reported TTTS with PABS cases, GA at FLP was 3 weeks earlier (17.7, IQR 16.6–19.2 weeks). It is noted that the severity and diagnostic GA of TTTS determine the timing at FLP, while postoperative PABS resulting from earlier intervention should be considered for the patients’ counseling and management.

Fetoscopic release is considered a potential therapeutic procedure for ABS without irreversible damage, as it can preserve the affected limb and its function and prevent fetal death in cases involving the umbilical cord. Its success has been reported in a few ABS cases in singletons, with good fetal outcomes. The reported success rate of fetal surgery in preserving limb function in ABS is as high as 75.7%, with few perioperative complications (15%) (8, 10). However, in TTTS, PABS antenatal detection was accurately reported in only 10 cases, of which 8 underwent fetoscopic release. Given the rarity of this condition, the efficacy of fetoscopic intervention for PABS remains to be established. The prognosis is widely variable and depends on the severity, affected body part and duration of the constriction. Umbilical cord involvement is a risk factor that is associated with IUFD when untreated (4, 17). Extremities are the most frequent anatomic sites involved and among cases of PABS in TTTS that did not receive treatment, 9.8% suffered amputation or necrosis of limbs and toes eventually. When diagnosed early, PABS is amenable to fetoscopic band release (14, 17). Similarly, in multiple pregnancy, we found that all PABS were fully recovered functionally without amputation after fetoscopic release of amniotic bands. To the best of our knowledge, case 1 we reported was the first case of fetoscopic release of PABS in TTTS affecting the recipient’s abdomen, which is relatively rare and successful. Although the fetoscopic release of the band with scissors or laser can save the affected body, postpartum plastic surgery may still be required for scar tissue repair (5, 9, 11).

The criteria and timing for performing fetal surgery to release the amniotic bands are important to mention. There is no consensus on the selection criteria for intrauterine intervention candidates in PABS. Hüsler et al. (21) presented a prenatal classification of ABS to standardize staging and management. Stage I consists of amniotic bands without signs of constriction; stage II of constriction without vascular compromise (compared with limb on opposite site), including stage IIA without or only mild lymphedema and stage IIB with severe lymphedema; stage III of severe constriction with progressive arterial compromise (vascular flow measurements distal and proximal to the constriction band), including stage IIIA with abnormal distal Doppler studies when compared to opposite limb and stage IIIB with no vascular flow to extremity, stage IV of bowing or fracture of a long bone at the constriction site; stage V of intrauterine amputation. Some researchers advised fetal surgery be seriously considered and discussed for ABS reaching stage III (10, 16).

The timing of the intervention must be balanced between the severity of the constriction and the risk of PPROM and preterm birth after intervention, which may occur in over half of the patients. Although Knijnenburg et al. (4) found no difference in PPROM between TTTS with or without PABS, PPROM (51.3%) and preterm birth (72%) remains the major risks in ABS after fetoscopic release (8, 14). In our review, among PABS cases in TTTS after FLP, PPROM occurred in 32.4% of those without fetoscopic release and in 66.7% of those with fetoscopic release. Post-procedural PPROM occurred in 51.3% (19/37) of ABS cases at a median onset of 28 days (range, 0–98 days) following fetoscopic release (8). Notably, among our reported cases of PABS in TTTS following a second fetoscopic release, this rate increased to 66.7% (4/6). Owing to the limited cases of PABS with fetoscopic release, it is unclear whether PPROM is a direct complication of the second fetoscopic procedure or is related to the intrinsic membrane properties. Therefore, it is essential to inform patients about benefits and risks of fetoscopic release of PABS.

However, this study has several limitations and potential biases. PABS is a rare complication. The review is focused and narrative rather than a formal systematic review; searches were restricted to PubMed and English-language reports, and publication bias is likely. Most of the studies consist of case reports and small series with some missing details (22–30). The pooled percentages should be viewed as descriptive signals only. Therefore, the results should be interpreted with caution. Reports of severe PABS are more likely to be published than those of mild cases, introducing a potential publication bias. It is very likely that there are several unpublished cases of unsuccessful fetal surgeries in PABS and the high success rate reported here may be an overestimation.

## Conclusion

Serial ultrasound examinations after invasive procedures in multiple pregnancies should focus on detecting signs of PABS. Antenatal detection of PABS is challenging but achievable. Given that PABS can lead to severe limb amputation or necrosis, fetoscopic release of the amniotic band is a technically feasible intervention in twin pregnancies and potentially beneficial in carefully selected prenatal diagnosed cases, but evidence remains limited and vulnerable to publication bias.

## Data Availability

The original contributions presented in this study are included in this article/Supplementary material, further inquiries can be directed to the corresponding author.
