# Supplementary material for: Case Report: Successful fetoscopic release of pseudoamniotic bands in twin-to-twin transfusion syndrome and twin reversed arterial perfusion sequence: report of two rare cases and review of the literature
Source: Front Med (Lausanne). 2026 Jul 17;13:1829018. doi: 10.3389/fmed.2026.1829018 (PMC13424010; doi:10.3389/fmed.2026.1829018)
Supplement: Supplementary file 1 [file Table_1.docx]

**Supplementary Table 1** Summary of the pseudoamniotic band syndrome cases in TTTS without intrauterine release.

| Author (Ref), year | Country | Cases, n | Quintero staging of TTTS | Placenta position | GA at laser, weeks | Timing of ABS detection | Affected fetus | Affected body part | Cord involvement | PPROM; GA, weeks | IUFD | GA at delivery, weeks | Postoperative follow-up and interventions |
| --- | --- | --- | --- | --- | --- | --- | --- | --- | --- | --- | --- | --- | --- |
| Rujiwetpongstorn et al. (22),2008 | Thailand | 1 | NA | NA | 24 | Postnatal | Recipient and donor | umbilical cord of recipient;  both legs and umbilical cord of donor | Both yes | No | Yes, donor | 31 | NA |
| Winer et al. (6), 2008 | France | 8 | 1 | Posterior | 21 | 2 cases of eight detected antenatally | Recipient | Two legs | No | No | Yes, donor | 26 | NA |
|  |  |  | 3 | Anterior | 23 |  | Recipient | Left arm | No | No | Yes, donor | 34 | NA |
|  |  |  | 3 | Anterior | 16 |  | Recipient | Right leg | No | Yes, PPROM between 25-33 | Yes, donor | 33.5 | NA |
|  |  |  | 2 | Anterior | 21 |  | Recipient | Right leg | No | Yes, PPROM between 25-33 | Yes, donor | 30 | NA |
|  |  |  | 3 | Anterior | 19 |  | Recipient | Right foot | No | Yes, PPROM between 25-33 | Yes, donor | 25.5 | NA |
|  |  |  | 1 | Posterior | 19 |  | Recipient | Left arm | No | No | Yes, donor | 33 | NA |
|  |  |  | 1 | Posterior | 20 |  | Recipient | Left arm | No | Yes, PPROM between 25-33 | No | 30 | NA |
|  |  |  | 3 | Posterior | 16 |  | Recipient | Right hand | No | Yes, PPROM between 25-33 | Yes, donor | 31 | NA |
| Karunaratne et al. (23),2011 | UK | 1 | NA | NA | 17 | Postnatal | Recipient | the left first and second toes, The right great toe, first and second toes | No | NA | No | 29 | NA |
| Rodrigues et al. (24), 2012 | Portugal | 1 | 2 | NA | 16.4 | Postnatal | Donor | upper left arm, the digits of both feet, with amputation of the distal phalange of the second digit of the right foot | No | No | Yes, recipient | 39.7 | A web reconstruction of the upper left arm was performed via Z-plasty on the 8th day of life |
| Shamshirsaz et al. (25), 2012^*^ | USA | 1 | 4 | NA | 16.6 | Postnatal | Recipient | Left big toe edema, amputation of the 2^nd^ and 3rd toes | No | No | No | 33.1 | NA |
| Ting et al. (5),2016 | Hongkong | 1 | 3 | Anterior | 18.3 | Postnatal | Recipient | Left hand | No | No | Yes, both | 26.1 | NA |
| Laffitte et al. (26),2017 | France | 2 | 2 | Posterior | 21.1 | Postnatal | Recipient | left side of the face, umbilical cord | Yes | Yes, PPROM27.6 | Yes, recipient | 27.6 | NA |
|  |  |  | 3 | Posterior | 16.1 | Postnatal | Recipient | Face, right hand, umbilical cord | Yes | No | Yes, recipient | 30.7 | NA |
| Li et al. (27),2019 | Taiwan | 2 | NA | NA | NA | Postnatal | Recipient | Amputation of right lower leg | NA | NA | Yes, donor | NA | NA |
|  |  |  | NA | NA | NA | Postnatal | Recipient | NA | NA | NA | No | NA | NA |
| Knijnenburg et al. (4),2020 | USA | 15 | NA | NA | 16.4 | Postnatal | Recipient | Left foot, digit I-V | No | NA | Yes, both | 22.6 | NA |
|  |  |  | NA | NA | 15.0 | Postnatal | Donor | Left lower leg, left foot digit II, III, right foot digit II-V | No | Yes | No | 29.7 | NA |
|  |  |  | NA | NA | 18.0 | Postnatal | Recipient | Right upper arm | No | No | No | 27.3 | NA |
|  |  |  | NA | NA | 17.7 | Postnatal | Donor | Toes | No | No | Yes, recipient | 33.6 | NA |
|  |  |  | NA | NA | 16.3 | Postnatal | Recipient | Right hand digit II | No | Yes | No | 30.6 | NA |
|  |  |  | NA | NA | 17.0 | Postnatal | Recipient | Left foot digit I-IV, right foot digit I, II, IV | No | No | No | 33.1 | NA |
|  |  |  | NA | NA | 17.9 | Postnatal | Recipient | Both feet digit II-IV | No | No | No | 29.3 | NA |
|  |  |  | NA | NA | 17.0 | Postnatal | Donor | Left foot digit I-III, right foot digit I-IV | No | No | No | 35.9 | NA |
|  |  |  | NA | NA | 17.0 | Postnatal | Recipient | Right foot digit II-IV | No | No | No | 36.4 | NA |
|  |  |  | NA | NA | 22.6 | Postnatal | Donor | Umbilical cord | Yes | No | Yes, donor | 34.2 | NA |
|  |  |  | NA | NA | 23.0 | Postnatal | Recipient | Left ankle | No | No | No | 36.0 | NA |
|  |  |  | NA | NA | 16.9 | Postnatal | Recipient | Right foot digit II-IV | No | No | No | 36.3 | NA |
|  |  |  | NA | NA | 15.0 | Postnatal | Donor | Right foot digit II-III | No | No | No | 38.6 | NA |
|  |  |  | NA | NA | 16.0 | Postnatal | Recipient | Right ankle | No | No | No | 30.6 | NA |
|  |  |  | NA | NA | 16.2 | Postnatal | Recipient | Right foot digit III, IV, right hand digit I, left arm | No | NA | No | 36.4 | NA |
| Rana et al. (28),2021^#^ | India | 1 | 4 | NA | 24.9 | Postnatal | Donor | right ankle | No | Yes, PPROM 29 | No | 29.3 | plastic surgery; 1- year follow- up, the right foot was normal both functionally and morphologically, a concentric contracture |
| Ogoyama et al. (29),2022 | Japan | 1 | 3 | NA | 17.6 | Postnatal | Recipient | Right ﬁngers (2-4), left toes (2-4), right index ﬁnger was necrotic | No | No | Yes, donor | 31.7 | 3 months of age, the right index ﬁnger was well epithelialized, and there was no functional disorder in the remaining digits |
| LeMoine et al. (30),2026 | USA | 8 | 3 | NA | 16.9 | Postnatal | Recipient | Arm | NA | Yes | No | 30 | No amputation |
|  |  |  | 1 | NA | 19.9 | Postnatal | Recipient | Arm | NA | No | No | 33.3 | No amputation |
|  |  |  | 3 | NA | 18.9 | Postnatal | Recipient | Arm, toes | NA | No | No | 30 | No amputation |
|  |  |  | 2 | NA | 18.4 | Postnatal | Donor | Toes (amputated) | NA | No | No | 32 | Digital amputation |
|  |  |  | 2 | NA | 19 | Postnatal | Donor | Toes (amputated) | NA | Yes | No | 26.7 | Digital amputation |
|  |  |  | 2 | NA | 16.9 | Postnatal | Donor | Shoulder, chest | NA | No | Yes, donor | 31.3 | No amputation |
|  |  |  | 3 | NA | 18.1 | Postnatal | Donor | Toes (amputated) | NA | No | No | 29 | Digital amputation |
|  |  |  | 2 | NA | 17.7 | Postnatal | Recipient | Arm | NA | Yes | No | 33 | No amputation |
| Summary |  | 42 cases and 43 afftected fetuses | Stage I: 17.4% (4/23 cases)  Stage II: 30.4% (7/23 cases)  Stage III: 43.5% (10/23 cases)  Stage IV: 8.7% (2/23 cases) | Posterior: 54.5% (6/11 cases)  Anterior: 45.5% (5/11 cases) | 17.7(16.6, 19.2) | Postnatal: 95.2% (40/42 cases)  Antenatal: 4.8% (2/42 cases) | Recipient: 71.4% (30/42 cases)  Donor:26.2% (11/42 cases)  Both twins: 2.4% (1/42 cases) | Upper limbs: 26.2% (11/42 fetuses)  Lower limbs: 54.8% (23/42 fetuses)  Both limbs: 9.5% (4/42 fetuses)  Only  umbilical cord: 4.8% (2/42 fetuses)  Face: 2.4% (1/42 fetuses)  Trunk: 2.4% (1/42 fetuses) | 15.2% (5/33 fetuses) | 32.4% (12/37 cases) | Total IUFD: 42.9% (18/42 cases)  Recipient IUFD: 9.5% (4/42 cases) Donor IUFD: 28.6% (  12/42 cases)  Both IUFD: 4.8% (2/42 cases ) | 31.0 (29.3, 33.5) |  |

* Monochorionic triamniotic triplet; NA, not available; Data are presented as median (interquartile range) or %(n/N). GA, gestational age; IUFD, intrauterine fetal demise; PPROM, preterm premature rupture of membranes; # selective fetal reduction of the recipient by bipolar cord coagulation;

**Supplementary Table 2.** Summary of the pseudoamniotic band syndrome cases in TTTS with intrauterine release.

| Author (Ref), year | Country | Cases, n | Quintero staging of TTTS | Placenta position | GA at laser, weeks | Timing of ABS detection | Affected fetus | Affected body part | Cord involvement | GA at release surgery | Technique | PPROM;GA,weeks | IUFD | GA at delivery, weeks | Postoperative follow-up and interventions |
| --- | --- | --- | --- | --- | --- | --- | --- | --- | --- | --- | --- | --- | --- | --- | --- |
| Ting et al. (5),2016 | Hongkong | 1 | 3 | Posterior | 18.9 | 25.1 | Recipient | Lift leg and right arm | Yes, donor | 26.4 | Nd-YAG | No | Yes, donor | 29 | Constriction rings, claw hand deformity requiring splintage at birth and neurolysis at 8 months of age with full functional recovery |
| Gueneuc et al. (10),2019 | France | 1 | NA | NA | NA | 22 | Recipient | Right foot; left lower extremity | No | 22.4 | Two ports (9 Fr and 10 Fr), Nd-YAG laser and scissors | NA | Yes, donor | 31.3 | No complications or constrictions in postpartum |
| Nassr et al. (12), 2020 | USA | 1 | 2 | NA | 18 | 21 | Recipient and donor | Right arm and umbilical cord of recipient;  Umbilical cord of donor | Both yes | 21 | Two ports (12 Fr), fetoscopic scissors and forceps | Yes, PPROM,26 | Yes, donor | 27 | Normal distal function |
| Bailleul et al. (11),2021 | France | 1 | 3 | NA | 18 | 22 | Recipient | Right foot | No | 22 | Fetoscopic scissors | NA | No | 31 | Stigma of constriction of the right foot but without edema or amputation |
| LeMoine et al. (30),2026 | USA | 3 | 3 | NA | 16.6 | NA | Recipient | Arm | NA | 27.4 | Two ports (9 Fr), fetoscopic scissors and forceps | Yes | No | 30.4 | No amputations and affected neurovascular function on postnatal exams |
|  |  |  | 3 | NA | 16.7 | NA | Recipient | Arm, finger | NA | 26.9 | Two ports (10 Fr), fetoscopic scissors and forceps | No | No | 32 | No amputations and affected neurovascular function on postnatal exams |
|  |  |  | 3 | NA | 17.6 | NA | Recipient | Arm | NA | 23.9 | Two ports (9 Fr), fetoscopic scissors and forceps | Yes | No | 24.7 | Gripping capabilities in the affected limb were decreased in  case 6 on postnatal exam |
| Our case | China | 1 | 2 | Posterior | 18.4 | 23 | Recipient | Abdomen | Yes | 23 | One port, fetoscopy,  scissors | Yes, PPROM, 34.9 | No | 34.9 | Surgical removal of the residual band and repair of the abdomen using Z-plasty after birth. |
| Summary |  | 8 cases, 9 affected fetuses | Stage II: 28.6% (2/7 cases)  Stage III: 71.4% (5/7 cases) | Posterior: 2/2 cases | 18 (16.6, 18.9) | 22 (21, 25.1) | Recipient: 87.5% (7/8 cases)  Both twins: 12.5% (1/8 cases) | Fetal limbs: 77.8% (7/9 fetuses)  Abdomen: 11.1% (1/9 fetuses)  Umbilical cord only: 11.1% (1/9 fetuses) | 66.7% (4/6 fetuses) | 23.5 (21, 27.4) | Two ports: 83.3% (5/6 cases)  One port: 16.7% (1/6 cases);  Nd-YAG laser: 12.5% (1/8 cases)  Scissors: 75% (6/8 cases)  Both laser and scissors: 12.5% (1/8 cases) | 66.7% (4/6 cases) | 37.5% (3/8 cases) | 30.7 (24.7, 34.9) |  |

NA, not available; Data are presented as median (range) or %(n/N). GA, gestational age; IUFD, intrauterine fetal demise; PPROM, preterm premature rupture of membranes; # selective fetal reduction of the recipient by bipolar cord coagulation;
